# Evaluation of Gluma® and MI Varnish™ for In-Office Treatment of Dentinal Hypersensitivity: An Observational Study

**DOI:** 10.7759/cureus.75530

**Published:** 2024-12-11

**Authors:** Rishabh Trivedi, Nayana Patel, Radha Vachhani, Nisha Verlianey, Jalpak Shukla, Mansi Sharma

**Affiliations:** 1 Department of Periodontology and Implantology, Government Dental College and Hospital, Jamnagar, Jamnagar, IND

**Keywords:** dentinal hypersensitivity, dentin desensitizers, gluma desensitizer, varnish, visual analog scale

## Abstract

Introduction

In their routine practice, dentists frequently encounter dentinal hypersensitivity, which is caused by the pulpal nerves' increased excitability due to fluid movement in the dentinal tubules. It is treated in-office using dentin desensitizers, which reduce hypersensitivity by obstructing the open tubules or desensitizing the free nerve endings present within the tubules. However, no substance or treatment plan has ever been proven to be the gold standard for the efficient treatment of dentinal hypersensitivity.

Aim

The aim of the study was to compare the effectiveness of two treatments for in-office dentinal hypersensitivity: Gluma^®^, a primer made of glutaraldehyde and hydroxyl ethyl methacrylate (HEMA), and MI Varnish™, a varnish made of 5% sodium fluoride and casein phosphopeptides.

Settings and design

This is an institutional, observational study.

Methods and material

A total of 19 patients with 64 teeth having dentinal hypersensitivity were observed. After isolation, tactile stimulus by an explorer, evaporative stimulus by a blast of air, and thermal stimulus by cotton soaked with propane-butane-isobutane were placed on the surface of the tooth, and the score was determined using the visual analog scale (VAS). The teeth were divided into groups (group A: Gluma; group B: MI Varnish) and respective desensitizer materials were applied, just after scaling and root planing and recording VAS scores. VAS scores were recorded immediately after scaling and root planing and at three and six weeks post-operatively. Paired t-test and Student's t-test (p<0.05) were used for statistical analysis.

Results

Both groups showed a significant reduction in VAS to all types of stimuli with time, compared to baseline (p<0.05). But at six weeks, patients in group B showed less increase in VAS score than did the patients in group A (p<0.05) for all types of stimuli.

Conclusions

MI Varnish was persistent and comparatively efficacious in reducing dentinal hypersensitivity than Gluma. But, longer durational clinical studies with larger sample sizes required to confirm the outcomes.

## Introduction

A dental condition known as dentinal hypersensitivity is described as "short, sharp pain arising from exposed dentin in response to stimuli typically thermal, evaporative, tactile, osmotic, or chemical and which cannot be attributed to any other form of dental defect or disease” [1]. The most widely recognized hypothesis to explain this occurrence is the traditional hydrodynamic theory, while other explanations have also been put forth. According to this, tooth sensitivity is caused by the nerves' increased excitability due to fluid movement in the tubules. The hydrodynamic process is the foundation of conventional methods for treating dental hypersensitivity [2].

By practicing non-surgical periodontal therapy, it is possible to effectively remove bacterial deposits from the tooth surface and treat periodontal disease. The cornerstone of non-surgical periodontal care is thought to be scaling and root planing. It may have a number of unfavorable side effects, including gingival recession and root dentin exposure, due to removal of cementum. Numerous dentinal tubules may be in contact with the oral environment, which would increase the patient's sensitivity to the root surface [3,4].

Numerous substances have been used to treat dentinal hypersensitivity for a long time, including potassium oxalate, ammonium hexafluorosilicate, propolis, and dentin bonding agents [5], but no substance or treatment plan has ever been proven to be the gold standard for the efficient treatment of dentinal hypersensitivity.

A commercially available desensitizing product called Gluma® (Heraeus Kulzer GmbH, Hanau, Germany) is made of glutaraldehyde and hydroxyethyl methacrylate (HEMA). HEMA occludes the dentinal tubules, whereas glutaraldehyde acts via coagulating amino acids and proteins in the dentin. HEMA's hydrophilic properties allow for deep penetration into dentinal tubules. Contrarily, HEMA's blocking effect is reversible and, with time, exposes the dentinal tubules [6]. The in-office therapy of MI Varnish™ (RECALDENT™, GC Corporation, Tokyo, Japan) operates as a calcium phosphate-based varnish by obstructing the dentinal tubules. The casein phosphopeptides (CPPs) in the varnish have a sodium fluoride content of 5%, which stabilizes the amorphous calcium phosphate phase so that it can provide bioavailable calcium, phosphate, and fluoride ions to the tooth surface [7].

Studies comparing fluoride varnishes are widely available in the literature; however, there is relatively little information on remineralizing varnishes used to treat dentinal hypersensitivity [8]. The aim of this study is to compare the effectiveness of two treatments for in-office dentinal hypersensitivity: Gluma, a primer made of glutaraldehyde and HEMA, and MI Varnish, a varnish made of 5% sodium fluoride and CPPs.

## Materials and methods

The study participants were chosen from patients who had dentinal hypersensitivity and visited the outpatient clinic. The Institutional Ethical Committee approved the procedures (clearance reference no. 02/01/2023(Ver.2.0)), and they were carried out in compliance with the Helsinki Declaration of 1975, as revised in 2000. In total, 68 teeth from 21 patients between the ages of 18 and 50 (11 females and 10 males) were used in the study, of which two male patients did not report after recording baseline scores. Therefore, 64 sites were observed for the study. The study was executed from May 2023 to September 2023.

Clinically and systemically healthy patients with dentinal hypersensitivity as their primary complaint before or after oral prophylactic procedures took part in the study. Patients who had received periodontal flap/regenerative therapy within the past six months, patients with deleterious oral habits such as smoking and tobacco chewing, pregnant and lactating mothers, individuals who have taken tooth-desensitizing medications in the last six weeks, and patients having allergies to milk and milk products (as CPP is derived from casein present in milk) were provided the alternative approaches to treat the dentinal hypersensitivity. Patients having teeth with caries, restorations, and wasting diseases were considered for the underlying etiology of dentinal hypersensitivity and treated accordingly.

**Figure 1:**
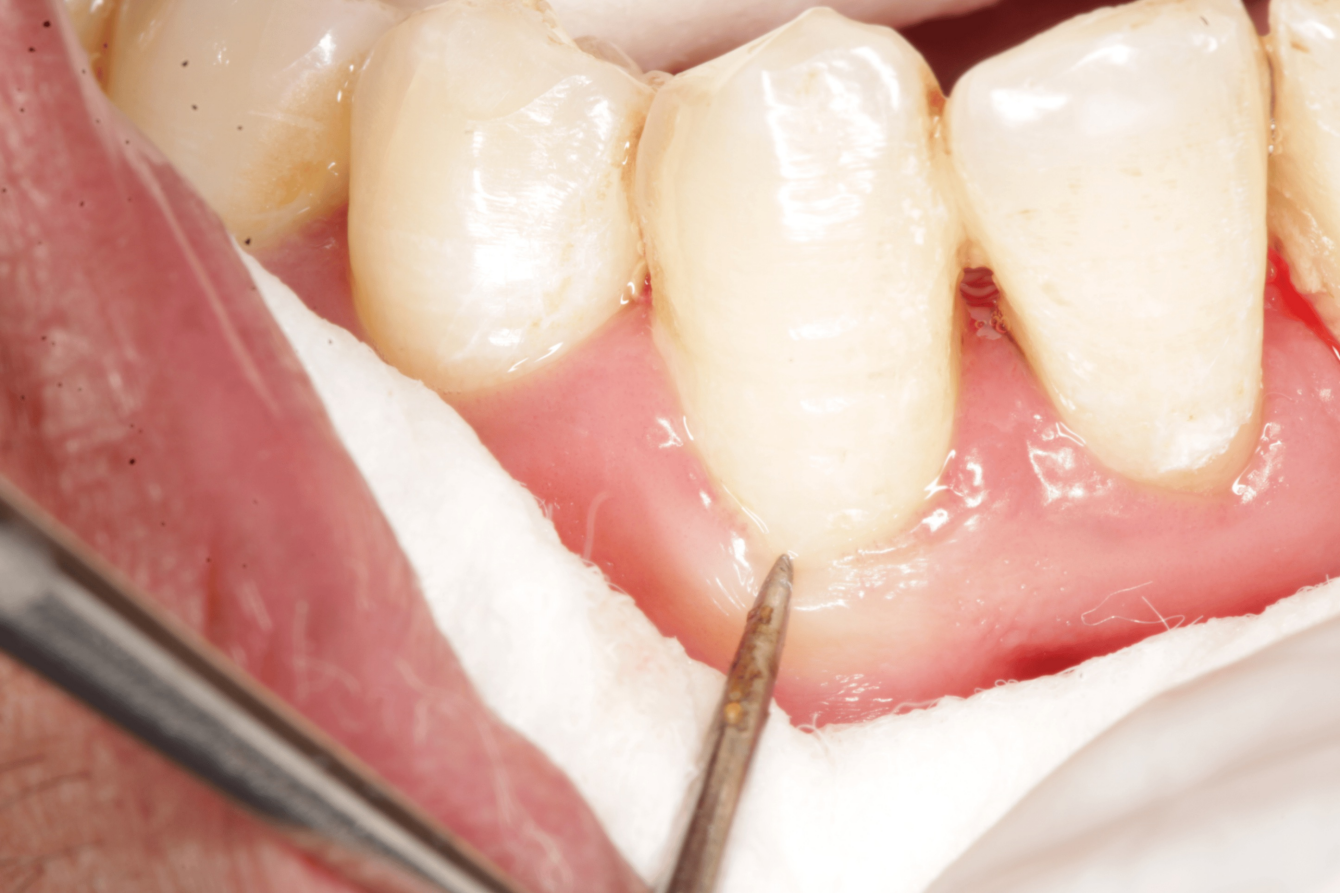
Assessment of visual analog scale using tactile stimulation

**Figure 2:**
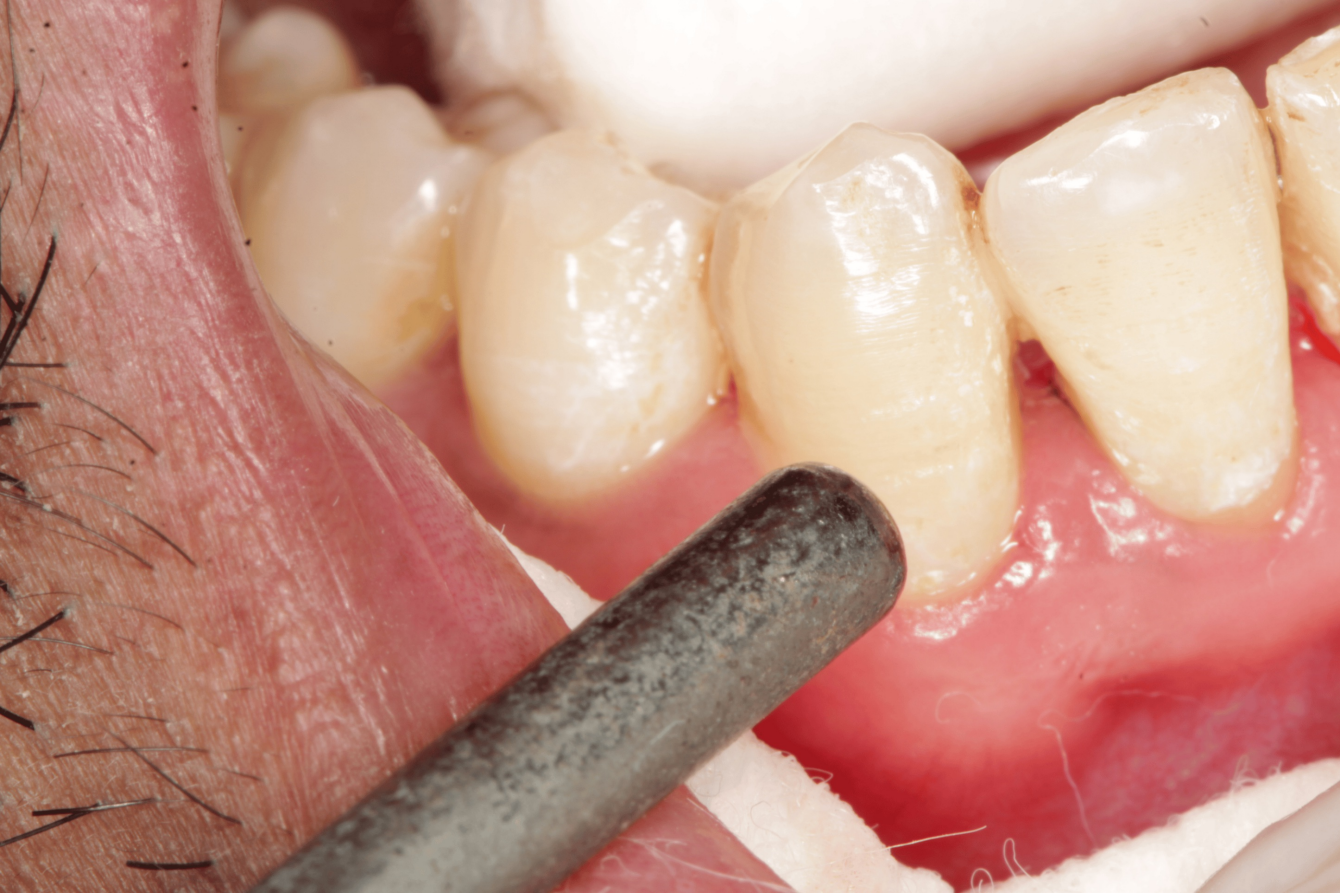
Assessment of visual analog scale using evaporative stimulus

**Figure 3:**
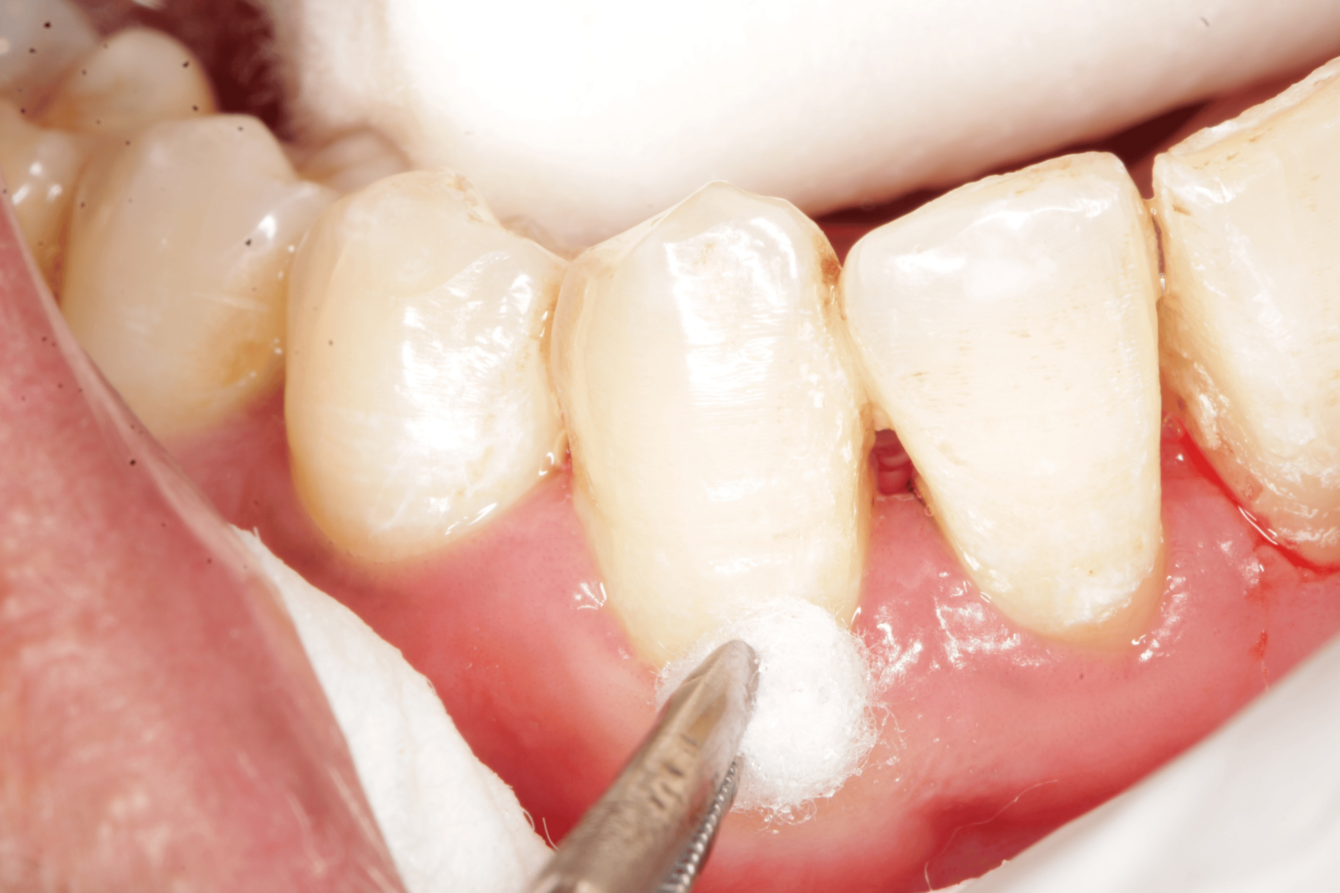
Assessment of visual analog scale using thermal stimulus

Only scaling and root planing were performed during the first appointment, and patients were given an explanation of the oral hygiene protocols. After a week, patients were reappointed for a follow-up, and baseline hypersensitivity values were noted. Dentinal hypersensitivity was assessed using a visual analog scale (VAS), which has a score range of 1 to 10, immediately after scaling and after three and six weeks [9]. The hypersensitivity scores via VAS were recorded using tactile stimulation by a dental explorer (Figure 1), evaporative stimulation by blasting air from the three-way syringe (Figure 2), and thermal stimulation by cotton containing propane-butane-isobutane at -50°C (Figure 3).

The VAS consists of a horizontal line with 10 cm of length and 10 scores along it. Score 0 denotes total painlessness (the extreme left end), whereas score 10 denotes the most excruciating pain (the extreme right end). After the tooth has been stimulated by the various stimuli described above, the participant specifies the intensity of pain perception. Given that it is assessed numerous times on the same patient, the VAS is thought to be a trustworthy instrument for evaluating the response. A minimum of 5 minutes should pass throughout the study time between the administrations of two distinct stimuli. When the discomfort reached an unacceptable level, the stimulus was cut off right away. Cotton rollers and a suction tool were used to isolate the teeth that would be evaluated. The score was then calculated using VAS (Figure 1) after a sharp dental explorer was run close to the cementoenamel junction of the respective teeth. After that, a burst of air was applied to the tooth surface from a distance of 0.5-1 mm and measured on a VAS (Figure 2) before measuring tooth sensitivity by applying cotton that had been soaked in propane, butane, and isobutane at a temperature of -50°C to the cementoenamel junction while the operator isolated the neighboring teeth with their fingers and cotton rolls (Figure 3). On each tooth, there was a minimum 5-minute gap between each stimulus.

**Figure 4:**
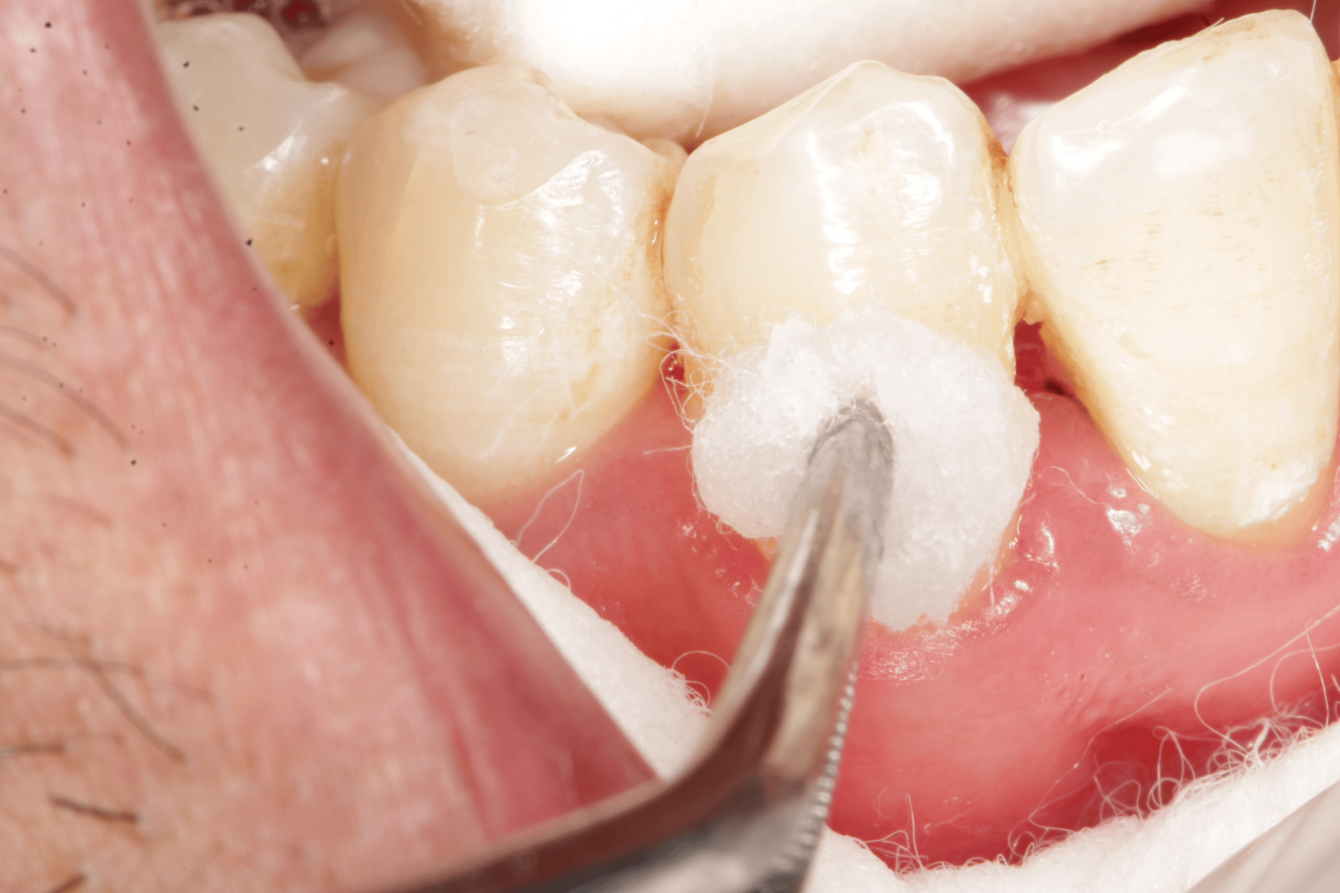
Gluma® applied using a small cotton pellet

**Figure 5:**
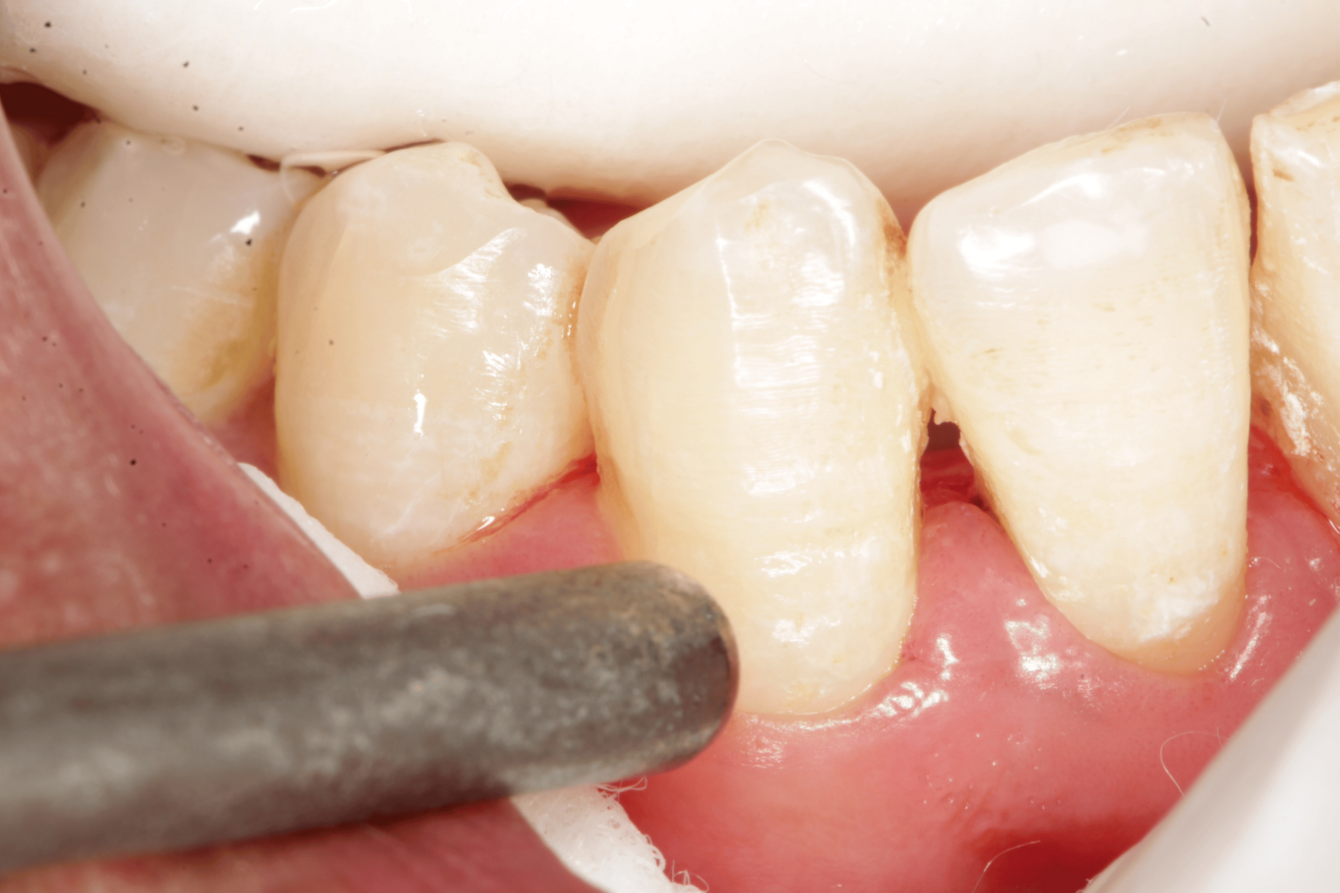
After application, extra material is removed using air blast

**Figure 6:**
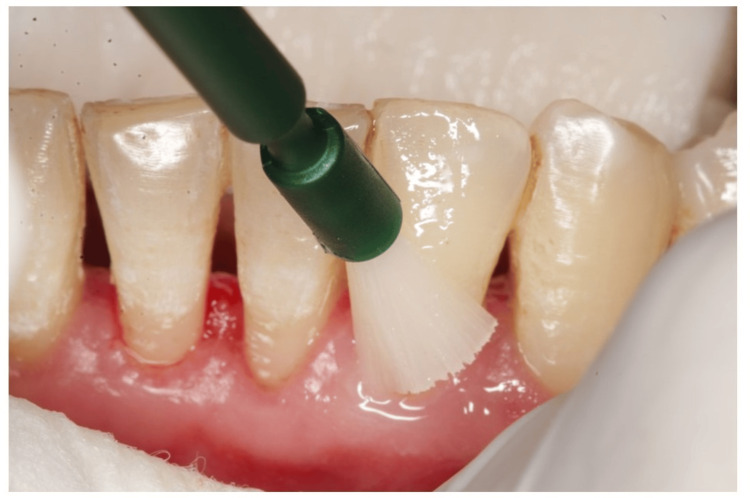
Application of MI Varnish

Sites with VAS > 2 values were divided into group A sites using Gluma (Figures 4, 5) and Group B sites using MI Varnish (Figure 6). Each site received a desensitizing agent according to the allotted group. The guidelines provided by the manufacturer were adhered to when applying the agents (Figures 4-6). A dry field was kept and contamination from water, and saliva was prevented. The patients were advised to refrain from eating anything sticky, hot, or hard, to abstain from alcohol-containing items (such as drinks and mouthwash), and to refrain from brushing and flossing their teeth for four hours [8]. Patients were instructed to brush their teeth twice a day with a soft-bristle toothbrush. During the research, patients were instructed to abstain from using any additional fluoride-containing toothpaste or mouthwash, but they were free to carry on with their regular oral care routines. At three and six weeks post-operatively, the sensitivity scores were recorded. At each appointment, the clinical outcome was assessed using the VAS scale, tactile stimulation, the air blast test, and the cold test [9].

Following the completion of all VAS tests, data were evaluated using the VAS mean and standard deviation. A paired t-test was used to compare the sensitivity levels within groups at various recall visits. Applying the Student's t-test, the scores of the two groups were intergroup compared at each time point. The cutoff for statistical significance was p<0.05.

## Results

A total of 21 patients gave consent for the study, and a total of 68 teeth were separated into two groups, i.e., group A (Gluma) and group B (MI Varnish). Out of 21 patients, 11 were females and 10 were males. The average age was 35.3 years. Two male patients did not come for three- and six-week follow-up visits, and thus four teeth were excluded from the study.

The intragroup comparison of VAS for all three stimuli of both groups is shown in Tables 1-3. For both groups, the response to all stimuli decreased from baseline to three weeks (p<0.05) and from baseline to six weeks (p<0.05). For both groups, the response to all stimuli increased from three weeks to six weeks (p<0.05), though VAS remained less at six weeks compared to baseline. However, for group A, this increase (three weeks to six weeks) was slightly more compared to that for group B.

**Table 1:** Intragroup comparison of visual analog scores for tactile stimulus *Statistical analysis was conducted using paired t-test, where a p-value of <0.05 was considered statistically significant SD, standard deviation

	Mean	SD	Mean	SD	p-Value
	Baseline		After 3 weeks		
Group A	6.09375	1.088336	2.09375	0.817525	<0.0001^*^
Group B	6.15625	1.297874	2.03125	0.822442	<0.0001^*^
	Baseline		After 6 weeks		
Group A	6.09375	1.088336	3.03125	0.646797	<0.0001^*^
Group B	6.15625	1.297874	2.625	0.707107	<0.0001^*^
	After 3 weeks		After 6 weeks		
Group A	2.09375	0.817525	3.03125	0.646797	<0.0001^*^
Group B	2.03125	0.822442	2.46875	0.841825	0.0004^*^

**Table 2:** Intragroup comparison of visual analog scores for evaporative stimulus *Statistical analysis was conducted using paired t-test, where a p-value of <0.05 was considered statistically significant SD, standard deviation

	Mean	SD	Mean	SD	p-Value
	Baseline		After 3 weeks		
Group A	6.3125	1.255632	2.28125	0.888434	<0.0001^*^
Group B	6.125	1.237844	2.125	0.870669	<0.0001^*^
	Baseline		After 6 weeks		
Group A	6.3125	1.255632	3.3125	0.692704	<0.0001^*^
Group B	6.125	1.237844	2.59375	0.756024	<0.0001^*^
	After 3 weeks		After 6 weeks		
Group A	2.28125	0.888434	3.3125	0.692704	<0.0001^*^
Group B	2.125	0.870669	2.59375	0.756024	0.0004^*^

**Table 3:** Intragroup comparison of visual analog scores for thermal stimulus *Statistical analysis was conducted using paired t-test, where a p-value of <0.05 was considered statistically significant SD, standard deviation

	Mean	SD	Mean	SD	p-Value
	Baseline		After 3 weeks		
Group A	6.15625	1.346666	2.375	0.793116	<0.0001^*^
Group B	6.53125	1.343668	2.34375	0.700662	<0.0001^*^
	Baseline		After 6 weeks		
Group A	6.15625	1.346666	3.40625	0.614837	<0.0001^*^
Group B	6.53125	1.343668	2.625	0.707107	<0.0001^*^
	After 3 weeks		After 6 weeks		
Group A	2.375	0.793116	3.40625	0.614837	<0.0001^*^
Group B	2.34375	0.700662	2.625	0.707107	0.0015^*^

The intergroup comparison of VAS for all three stimuli of both groups is shown in Table 4. For all the stimuli, there was no statistically significant difference between the two groups at baseline and three weeks (p>0.05). But at six weeks for all the stimuli, the data collected from group A were slightly higher than that from group B (p<0.05).

**Table 4:** Intergroup comparison of visual analog scores for all three stimuli between both the groups at baseline and at three weeks and six weeks *Statistical analysis was conducted using unpaired t-test, where a p-value of <0.05 was considered statistically significant SD, standard deviation

		Group A		Group B		p-Value
		Mean	SD	Mean	SD	
Tactile stimulus	Baseline	6.09375	1.088336	6.15625	1.297874	0.8353
	After 3 weeks	2.09375	0.817525	2.03125	0.822442	0.7615
	After 6 weeks	3.03125	0.646797	2.46875	0.841825	0.0039^*^
Evaporative stimulus	Baseline	6.3125	1.255632	6.125	1.237844	0.5497
	After 3 weeks	2.28125	0.888434	2.125	0.870669	0.48
	After 6 weeks	3.3125	0.692704	2.59375	0.756024	0.0002^*^
Thermal stimulus	Baseline	6.15625	1.346666	6.53125	1.343668	0.2691
	After 3 weeks	2.34375	0.787375	2.375	0.793116	0.8748
	After 6 weeks	3.40625	0.614837	2.625	0.707107	<0.0001^*^

## Discussion

The main objective of treating dentinal hypersensitivity is to either physically or chemically occlude the dentinal tubules or desensitize the nerves by depolarizing the nerve terminal's cellular membrane. Resin materials have reportedly been effective in lowering dentin hypersensitivity. The goal of using resins to treat dentin hypersensitivity is to physically close the open dentin tubules and totally seal orifices by making resin plugs or resin tags [10].

One of the most effective desensitizing agents is Gluma desensitizer, which contains HEMA and glutaraldehyde. HEMA is a hydrophilic monomer that can physically obstruct the dentinal tubules by penetrating acid-etched and moist dental hard tissues [11]. By causing the plasma proteins in the dentinal tubules to coagulate, glutaraldehyde, a potent fixative or flocculating agent, creates a physiological seal [12]. It has been demonstrated that the combination of HEMA and glutaraldehyde can occlude dentinal tubules 50-200 μm deep [6,13].

CPP-amorphous calcium phosphate (CPP-ACP) with 2.26% (w/w) fluoride is a component of MI Varnish. ACP localized to the tooth structure serves as a calcium phosphate reservoir and raises the level of calcium phosphate in plaque. Additionally, buffering the free calcium and phosphate ion activity keeps dental enamel supersaturated. These ions transform into stable crystalline phases, such as octacalcium phosphate or apatitic byproducts [14]. The presence of bioavailable CPP-ACFP nanocomplexes is compatible with the MI Varnish's release of fluoride, calcium, and inorganic phosphate ions in addition to CPP. These nanocomplexes feature electroneutral ion clusters and a hydrodynamic radius of 2.1-0.26 nm and diffuse quickly through intraprismatic voids from the varnish into the enamel subsurface [7].

Studies have demonstrated that, despite being subjective, the VAS scale is reliable for capturing patient reactions. An air blast was employed in the study because it is thought to be the most effective approach to get the patient to respond. It comprises a brief blast from a dental unit three-way syringe that is directed 0.5 cm from the exposed buccal cervical root surface and has a pressure range of 40-65 psi and a temperature range of 14°C-26°C. Other studies of dental sensitivity have employed this methodology [15]. It has been suggested that at least two stimuli be employed, the least intense one coming first and then the second one, with little to no interaction between the two. As such, three stimuli were used in this investigation, and a 5-minute break was suggested between the two assessments [8].

As the previous studies on MI Varnish were conducted either for a very short period, i.e., two weeks [16], one week [8], or in vitro [7,16], a study period of six weeks was considered for this study. In the present study, there was a continuous decline in the VAS of all stimuli for both groups from baseline to three weeks, as it concurs with previous studies for these materials [6,12-14,16]. But from three to six weeks, the VAS slightly increased for all stimuli in both groups. This increase was higher in group A compared to group B. This VAS increase for group A (Gluma) was in accordance with a previous study conducted by Al-Qahtani in 2019 [17].

Since there was no statistically significant difference for VAS at baseline in any of the stimuli in both groups, it was certain that there was no difference in the type or intensity of pain in patients of both groups. The intergroup comparison shows no difference in the VAS of any stimuli between the groups at three weeks, which means both the materials have an equal amount of pain reduction at this time point. At six weeks, group A showed a statistically significant higher gain in VAS for all stimuli than group B, which means MI Varnish has a more persistent and comparatively higher effect than Gluma in reducing dentinal hypersensitivity. To explain, the combination of CPP-ACP and F to form CPP-ACFP nanocomplexes has been shown to be superior to F alone in inhibiting enamel demineralization and promoting remineralization, and the presence of the CPP-ACFP nanocomplexes would explain the superior ability of MI Varnish to inhibit demineralization in this study [7].

Since this study is just an observational study, randomization and blinding were not performed. Therefore, there are chances of selection bias. However, all the efforts were made to provide the best treatment options to the patients. Given that this is a six-week follow-up study, the authors acknowledge that additional research with a longer observation period and sound design is necessary to assist in ascertaining how frequently the materials need to be reapplied.

## Conclusions

To conclude, both materials showed almost equal amounts of reduction in dentinal hypersensitivity. But at six weeks, MI Varnish was persistent and comparatively efficacious in reducing dentinal hypersensitivity than Gluma desensitizer. Hence, given the chance, further longer durational studies with larger sample sizes are needed to further support this conclusion. Furthermore, although more research is required, the authors acknowledge that restorative materials can still be used to control dentinal hypersensitivity, especially in situations where varnishes and desensitizing agents have not worked or have only temporary advantages.
